# Lung ultrasound is a reliable diagnostic technique to predict abnormal CT chest scan and to detect oxygen requirements in COVID-19 pneumonia

**DOI:** 10.18632/aging.104150

**Published:** 2020-10-30

**Authors:** Géraldine Falgarone, Frédéric Pamoukdjian, Johann Cailhol, Audrey Giocanti-Auregan, Sandrine Guis, Guilhem Bousquet, Olivier Bouchaud, Olivier Seror

**Affiliations:** 1 Université Sorbonne Paris Nord, INSERM UMR_S942, Bobigny F-93009, Paris, France; 2 AP-HP, Hôpital Avicenne, Unité de Médecine Ambulatoire (UMA), Bobigny F-93009, Paris, France; 3 AP-HP, Hôpital Avicenne, Service de Médecine Gériatrique, Bobigny F-93009, Paris, France; 4 AP-HP, Hôpital Avicenne, Service de Maladies Infectieuses et Tropicales, Bobigny F-93009, Paris, France; 5 Université Sorbonne Paris Nord, EA 3412, Bobigny F-93009, Paris, France; 6 AP-HP, Hôpital Avicenne, Service d’Ophtalmologie, Bobigny F-93009, Paris, France; 7 Service de Rhumatologie, AP-HM, Aix-Marseille Université, Marseille, France; 8 CRMBM/CEMEREM, UMR CNRS 7339, Aix-Marseille Université, Marseille, France; 9 AP-HP, Hôpital Avicenne, Service d’Oncologie Médicale, Bobigny F-93009, Paris, France; 10 AP-HP, Hôpital Avicenne, Service de Radiologie Interventionnelle, Bobigny F-93009, Paris, France; 11 Université Sorbonne Paris Nord, INSERM UMR_S1162 Paris, Bobigny F-93009, Paris, France; 12 Inserm U1138, Centre de Recherche des Cordeliers, Paris F-75006, Paris, France

**Keywords:** COVID-19, pneumonia, lung ultrasound, oxygen need, diagnosis, follow-up

## Abstract

COVID-19 pneumonia can be severe, with an unpredictable evolution and high mortality prevalence in older patients. The diagnosis is usually performed by RT-PCR or CT chest scan. Lung ultrasonography (LUS) has been proposed as an alternative method to monitor patients with COVID-19 pneumonia.

To assess the diagnostic performance of LUS, we performed LUS using a portable device and adapting a protocol already used in Acute Respiratory Syndrome. We used the score obtained with the index we created to assess for LUS diagnostic performance as compared to lung CT chest scan and to predict for oxygen requirements.

Daily bedside LUS was easy to perform and microbiologically safe. LUS was 89% sensitive and 100% specific in predicting CT chest scan abnormalities, and 95% sensitive and 67% specific in detecting oxygen requirements.

This is the first report on the diagnostic performance of LUS as compared to CT chest scan for the diagnosis of COVID-19 pneumonia and assessments of oxygen requirements by LUS. LUS could help in the orientation of dyspneic patients to intensive care. It could also be proposed when there is limited access to CT scan in the context of a pandemic crisis, or to implement clinical lung examinations for outpatient follow-up.

## INTRODUCTION

COVID-19 pneumonia can be severe and its evolution unpredictable [1, 2] with a high mortality prevalence of 26% in older inpatients [3]. Computed Tomography (CT) chest scan is the imaging "gold standard" to detect COVID-19 pneumonia, in particular when RT-PCR is negative [4, 5]. In the dramatic context of a pandemic situation, access to CT chest scan can be limited, and repeated CT scans, if an imaging follow-up is required, can be difficult to perform due to costs, invasiveness and infectious risks. In intensive care units, lung ultrasonography (LUS) has proved to be very helpful in managing acute respiratory distress syndrome, (ARDS) [6, 7], and has recently been suggested to manage COVID-19 pneumonia [8–10]. To date no study has yet assessed the diagnostic performance of LUS in COVID-19 as compared to CT chest scan. Furthermore, this information is of major importance in the event of a second wave, to manage patient flows, or in emerging infectious lung diseases. Here we propose for the first time a practical diagnostic process using bedside LUS with a portable device which can be used for both diagnosis and daily monitoring of patients with COVID-19 pneumonia.

## RESULTS

### Characteristics of patients and diagnostic performances of LUS in detecting lung damage observed on CT chest scan in COVID-19

The mean age of the 50 consecutive patients with confirmed COVID-19 was 54.3 years, 34% were older than 65 years, 74% were male and 74% overweight with a median BMI = 28 kg/m². They had damage detected on CT scan described as minor (10%), moderate (38%), extensive (46%) or severe (6%). 72% of all patients had a positive RT-PCR for SARS-Cov-2. 80% had at least one significant comorbidity, 30% had two comorbidities, and 15% had three comorbidities (hypertension, diabetes, overweight). The prevalence of hypertension was 28%, and the prevalence of diabetes was 28% (Table 1).

**Table 1:** Baseline characteristics of 50 consecutive inpatients with COVID-19.

**Variables**	**Mean ± SD or Median (Q1-Q3)**	**N (%)**
Age (y)	54.3 ± 17.3	
Gender (men)		37 (74)
US-index	0.40 ± 0.10	
Number of evaluated Locations (% in the cohort)		
Front Upper Right		50 (100)
Front Lower Right		49 (98)
Front Upper Left		49 (98)
Front Lower Left		44 (88)
Side Upper Right		39 (78)
Side Medium Right		34 (68)
Side Lower Right		20 (40)
Posterior Right		37 (74)
Side Upper Left		42 (84)
Side Medium Left		33 (66)
Side Lower Left		22 (44)
Posterior Left		37 (74)
		
Time lapse between symptoms and LUS (days)	9 (7-14)	
Time lapse between CT chest scan and LUS (days)	1.5 (1-6.5)	
CT chest scan damage		
None		3 (6)
Mild		2 (4)
Moderate		19 (38)
Extensive		23 (46)
Severe		3 (6)
Sars-cov-2 RT-PCR		
Positive		36 (72)
Negative		11 (22)
Not performed		3 (6)
Oxygen therapy		
Consumption > 0 l/min (yes)		41 (82)
Requirement (l/min)	2.5 (1-4)	
BMI (kg/m^2^)	28 (25.1-30.6)	
Prevalence of comorbidities:		
Number of comorbidities	1 (1-2)	
1 at least		40 (80)
2 comorbidities		12 (24)
3 comorbidities		6 (12)
Overall HTA prevalence		14 (28)
Overall Diabetes prevalence		14 (28)

US: Ultrasound; BMI: Body Mass Index; HTA: hypertension.

### Severity index for lung involvement based on LUS

**Figure 1:**
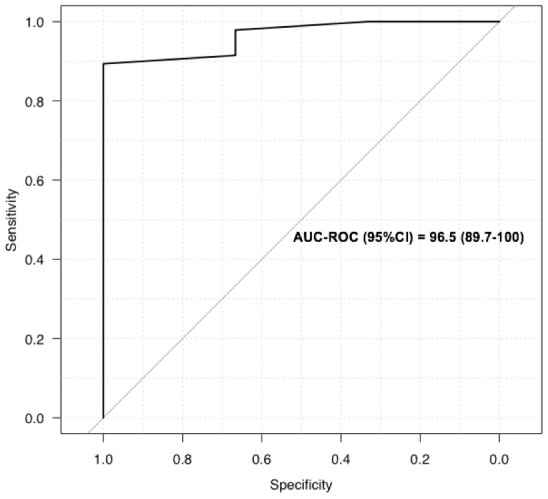
**Receiver operating characteristics curves for LUS to screen for COVID-19: ROC for LUS to predict abnormal CT scan.**

**Figure 2:**
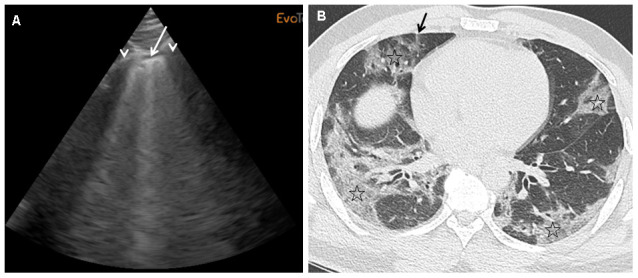
(**A**)This panel shows a grade 2 LUS image for a 45-year-old man at 10 days from initial symptoms, requiring oxygen (6 l/min), with a severity index of 0.58. On this sagittal ultrasound view encompassing a right anterior inter-rib space (arrows: rib shadows), one can see a typical lung rocket (arrow) as breath moving comet-tail artefact. (**B**) This panel shows an axial image of the corresponding CT scan performed at admission with typical COVID-19 interstitial pneumonia combining septal thickening (arrow) and peripheral ground glass opacities (stars).

We performed daily bedside LUS evaluations using a portable device to evaluate COVID-19 pneumonia. LUS evaluations of lung damage were graded in the six categories described for ARDS, in a blinded manner for oxygen needs and CT scan grading, based on a twelve-point grading system (see Materiel and Methods section). We designed this severity index that is based on evaluations done with certainty to be able to take into account missing data and feasibility (see Material and Methods section for details). Table 2 reports the LUS scoring taking into account missing data, feasibility due to anatomical or posture problems, and oxygen requirements for the first 12 patients included. For the totality of the patients, the mean LUS severity score was 0.40 (ranging from 0.2 to 0.63). LUS exhibited 89% sensitivity and 100% specificity in predicting an abnormal CT scan with an AUC of 96% (95% CI) (Figure 1). When our LUS severity index was tested at different thresholds, we found that the value 0.32 was robust across sensitivity analyses, providing the optimal index to predict CT scan severity (Supplementary Table 1). Figure 2 illustrates the case of a 45-year-old man at 10 days from initial symptoms with severe pneumonia on CT scan. The LUS severity index was 0.58, with 10 locations scored from 1 to 4.

**Table 2:** Examples of LUS scoring for the first 12 patients.

	**Location 1**	**Location 2**	**Location 3**	**Location 4**	**Location 5**	**Location 6**	**Location 7**	**Location 8**	**Location 9**	**Location 10**	**Location 11**	**Location 12**	**Nb Locations**	**Max obtainable**	**Score value**	**02 need (l/mn)**
pt#1	1	1	1	2	1	1	4		1	3	3		10	50	0.36	1
pt#2	1	1	1	1				3	1	1		4	8	40	0.33	1.5
pt#3	1	2	3	2	2	2		2	4	1		4	10	50	0.46	4
pt#4	1	1	3	3	3	3							6	30	0.47	8
pt#5	1	1	1	1	1			1	1		3	4	9	45	0.31	0
pt#6	1	1	1	1	1	3		3	3	3	3	3	11	55	0.42	4
pt#7	1	2	1	1	3	3		4	1			4	8	40	0.50	9
pt#8	3	3	1	1	4	4	4		3	3	3		10	50	0.58	6
pt#9	1	1	1	1	1	1	4	2	1	2	2	3	12	60	0.33	4
pt#10	1	2	1		3	3	4	1	1	1	1	2	11	55	0.36	3
pt#11	1	2	1	2	2	3	3	3	2	2		3	11	55	0.44	1
pt#12	1	1	1	2	3	3		4	2	3		4	10	50	0.48	4

pt: patients. No. locations: Number of locations evaluated with certainty. Max: maximum obtainable, which is the number of locations multiplied by 5. Score value: Sum of location values divided by Max.

### Diagnostic performance of LUS in detecting oxygen requirements in COVID-19 pneumonia

**Figure 3:**
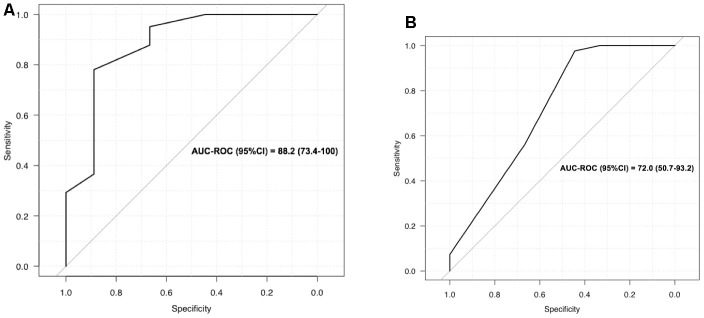
(**A**) LUS to screen for oxygen requirement ≥ 0.5 l/min; (**B**) CT scan to screen for oxygen requirement ≥ 0.5 l/min.

LUS was a good screening test for oxygen requirements (0.5 litre/min or more) with 95% sensitivity, 67% specificity, and an AUC of 88% (95% CI) (Figure 3A). The best LUS severity index threshold for oxygen therapy was 0.32 (Table 2) because the PPV was 92.9 (80-98) and the NPV 75.0 (35-97), with a diagnostic accuracy of 90.0 (78-97). In contrast, on the CT scans of our 50 patients, global scoring was not as reliable in predicting oxygen requirements, with an AUC of 72 (CI: 50-93%) (Figure 3B). For example, a score of 2 (moderate lung damage of 10 to 25%) had sensitivity of 98% but specificity of 44%. The LUS diagnostic performances were also robust in the sensitivity analyses according to BMI, and thus reliable for overweight patients, since the PPV was still 100% for CT scan scoring, while it varied for oxygen requirements, ranging from 93% to 91% and 95% for the whole cohort, normal weight, and over weighted patients respectively (Table 3).

**Table 3:** Diagnostic performances of the LUS - severity index in detecting oxygen requirements ≥ 0.5 l/min.

**LUS-index**	**Se (%) 95%CI**	**Sp (%) 95%CI**	**PPV (%) 95%CI**	**NPV (%) 95%CI**	**Diagnostic accuracy (%) 95%CI**
**Whole cohort (n=50)**					
≥ 0.30	100 (87.4-100)	44.4 (13.7-78.8)	89.1 (76.4-96.4)	100 (28.4-100)	90.0 (78.2-96.7)
≥ 0.31	97.6 (87.1-99.9)	55.6 (21.2-86.3)	90.9 (78.3-97.5)	83.3 (35.9-99.6)	90.0 (78.2-96.7)
**≥ 0.32**	**95.1 (83.5-99.4)**	**66.7 (29.9-92.5)**	**92.9 (80.5-98.5)**	**75.0 (34.9-96.8)**	**90.0 (78.2-96.7)**
≥ 0.33	87.8 (73.8-95.9)	66.7 (29.9-92.5)	92.3 (79.1-98.4)	54.5 (23.4-83.3)	84.0 (70.9-92.8)
≥ 0.34	82.9 (67.9-92.8)	77.8 (40.0-97.2)	94.4 (81.3-99.3)	50.0 (23.0-77.0)	82.0 (68.6-91.4)
**BMI ≤ 28 kg/m2 (n=26)**					
0.30	100 (78.1-100)	50.0 (6.80-93.2)	91.7 (73.0-99.0)	100 (9.40-100)	92.3 (74.9-99.1)
0.31	95.5 (77.2-99.9)	50.0 (6.80-93.2)	91.3 (72.0-98.9)	66.7 (9.40-99.2)	88.5 (69.8-97.6)
**0.32**	**90.9 (70.8-98.9)**	**50.0 (6.80-93.2)**	**90.9 (70.8-98.9)**	**50.0 (6.80-93.2)**	**84.6 (65.1-95.6)**
0.33	81.8 (59.7-94.8)	50.0 (6.80-93.2)	90.0 (68.3-98.8)	33.3 (4.30-77.7)	76.9 (56.4-0.91)
**BMI > 28 kg/m2 (n=24)**					
0.30	100 (75.1-100)	40.0 (5.30-85.3)	86.4 (65.1-97.1)	100 (9.40-100)	87.5 (67.6-97.3)
0.31	100 (75.1-100)	60.0 (14.7-94.7)	90.5 (69.6-98.8)	100 (19.4-100)	91.7 (73.0-99.0)
**0.32**	**100 (75.1-100)**	**80.0 (28.4-99.5)**	**95.0 (75.1-99.9)**	**100 (28.4-100)**	**95.8 (78.9-99.9)**
0.33	94.7 (74.0-99.9)	80.0 (28.4-99.5)	94.7 (74.0-99.9)	80.0 (28.4-99.5)	91.7 (73.0-99.0)

Se = sensitivity; Sp = specificity; PPV = positive predictive value; NPV = negative predictive value; Bold = Best threshold.

## DISCUSSION

In this study, we have shown the feasibility of LUS for the diagnosis of pneumonia and oxygen requirements in the context of the COVID-19 pandemic. LUS is feasible in COVID-19 units with their high infectious risk, particularly with a portable device. Safety conditions were fulfilled, applying specific COVID-19 hygiene procedures. Operators were wearing adequate protection and as the device was restricted to the COVID-19 unit, the operator had to simply clean the probe, the wire, and the LUS device itself after each examination. From the technical method described by Soldati et al (7), we developed a scoring system that is easy to perform and reliable for the diagnosis of COVID-19 pneumonia, whatever the patient’s position. The proposed LUS severity index was calculated by summing all scores for unequivocal locations, and then dividing the sum by the maximum obtainable (the maximum being 60 for the 12 locations evaluated). The robustness of our assessment is related to the choice we made to only take into account scores allocated with certainty. A striking result was that the prediction of oxygen therapy requirements was excellent for LUS but poor for CT chest scan, which is considered the gold standard for COVID-19 pneumonia diagnosis. However, the discordance between CT scan and clinical severity has already been reported [11]. The optimal threshold of 0.32 for the severity index could be appropriate to motivate increase in oxygen therapy, and to prevent inappropriate early reduction. This could be of major interest in the follow-up of patients with COVID-19 pneumonia, since LUS can easily be performed daily at the bedside. For severe COVID-19 inpatients, the use of LUS could be helpful to comfort the estimation of oxygen needs at hospital discharge. As a perspective following these observations, LUS could also be proposed for outpatients, particularly older patients since COVID-19 is liable to impinge upon the follow-up of chronic diseases (autoimmune diseases, cancers, chronic obstructive pulmonary disease). LUS could be useful in diagnosis, as well as in follow-up of lung diseases in an ultrasonoscopy procedure where lung auscultation is performed or replaced by lung visualisation with ultrasound [9], especially for patients that need low irradiation techniques like pregnant women.

In addition, LUS could also be of particular interest when CT chest scan access is limited, or in the case of so-called re-infestation/reactivation of COVID-19 to diagnose pneumonia (16).

## CONCLUSIONS

This proof-of-concept study proposing an original scoring system and a severity index requires confirmation by larger multicentre studies, which can now benefit from a clearly described, easy procedure. This would help in evaluating lung disease using ultrasound in a clinical practice also named ultrasonoscopy.

## MATERIALS AND METHODS

We followed the Standards for Reporting Diagnostic Accuracy studies (STARD) recommendations [12].

### Patients

From 04/03/2020 to 04/14/2020, 50 consecutive patients with COVID-19 pneumonia admitted to the department of infectious diseases in Avicenne hospital, Paris, were systematically evaluated with LUS. Baseline clinical characteristics were prospectively collected, including gender, age, time lapse from onset of COVID-19 disease, and major comorbidities as previously defined [13], Body Mass Index (BMI) before the illness, CT chest scan severity index (standard national recommendation, based on [14]), SARS-Cov-2 RT-PCR test result, C-reactive protein level (mg/l), lymphocyte count as an indicator of viral disease severity [15], and oxygen therapy requirements (litre/min). Patient consent was collected in accordance with national ethics rules.

### Daily bedside evaluation using LUS

LUS evaluations were performed by a single operator experienced in articular ultrasonography, using a portable device (ExoTouch, Quantel Medical ®, Cournon d’Auvergne, F-63808 France), and a 3,5 to 6 Mhz convex array transducer (CC560A, ExoTouch, Quantel Medical ®, Cournon d’Auvergne, F-63808 France). LUS evaluations of lung damage were graded in the six categories described for ARDS [16]: normal pleural image with A lines was graded as 1, B lines with lung rockets grade 2, ground glass rockets grade 3, condensation grade 4, and neo organization known as hepatisation grade 5. Pleurisy, theoretically graded 6, was not found among our patients. LUS was performed blind to oxygen needs and CT scan grading, and results were expressed on a twelve-point grading system with 3 key features defining our process 1) the patient’s position was not limited since we performed LUS in lying, siting, or standing position; 2) the anatomical location of the examination, with, for each hemi thorax, 2 anterior locations, 3 side locations, and 1 posterior location (annex 1); 3) the grading system from 1 to 5 as described above. We decided that only unequivocal scores were to be collected. Uncertain scores were simply considered as non-available, whatever the reason (position, anatomical variation, technical difficulties). Thus, the number of available measures defined the feasibility of LUS across the 12 locations assessed.

### Development of a severity index for lung damage based on LUS

To provide a reproducible method using LUS, we created a severity index for lung damage as follows: the sum of the scores obtained for each measurement location was divided by the maximum possible score based on evaluations done with certainty. For example, when a measure was obtained only for 8 locations, the maximum score was 5 x 8 = 40. In contrast, when a measure was obtained for all 12 locations, the maximum score was 5 x 12 = 60. If the sum of the LUS evaluation is 20, in the first case, with a total of 8 locations collected with certainty, the index of severity index is 0.5 (i.e. 20/40). In the second case, where all locations were assessed with certainty, the severity index is 0.33 (i.e. 20/60). Thus this index takes missing data and feasibility into account.

### Statistical analysis

Qualitative variables were expressed as numbers and proportions, and quantitative variables as means and standard deviation (SD) or medians and quartiles (Q1-Q3) as appropriate. Diagnostic performances were assessed on the basis of sensitivity (Se), specificity (Sp), positive predictive value (PPV), negative predictive value (NPV), positive likelihood ratio (PLR), and negative likelihood ratio (NLR). The diagnostic accuracy of LUS in detecting lung damage on CT chest scan or oxygen requirements was quantitatively analysed by the area under the receiver operating characteristic curve (AUC-ROC) with 95%CI, and graphically from the ROC curve. Sensitivity analyses were performed to test the robustness of the results by 1) varying the thresholds of the LUS-index (0.30, 0.31, 0.32, 0.33, and 0.34); and 2), using BMI as a function of the median of 28 kg/m^2^. All tests were two-sided, with *P*<0.05 considered statistically significant. The data was analysed using R statistical software (version 3.4.3, R Foundation for Statistical Computing, Vienna, Austria; http://www.rproject.org).

## Supplementary Material

Supplementary Table 1
