# Supplementary material for: Lung ultrasound is a reliable diagnostic technique to predict abnormal CT chest scan and to detect oxygen requirements in COVID-19 pneumonia
Source: Aging (Albany NY). 2020 Oct 30;12(20):19945–53. doi: 10.18632/aging.104150 (PMC7655154; doi:10.18632/aging.104150)
Supplement: Supplementary Table 1 [file aging-12-104150-s001..pdf]

SUPPLEMENTARY TABLE

Supplementary Table 1. Diagnostic performances of the LUS- severity index to predict CT chest scan severity.

US-index	Se (%) 95%CI	Sp (%) 95%CI	PPV (%) 95%CI	NPV (%) 95%CI	PLR 95%CI	NLR 95%CI	Diagnostic accuracy (%) 95%CI
**Whole cohort (n=50)**							
0.30	95.7 (85.5-99.5)	66.7 (9.40-99.2)	97.8 (88.5-99.9)	50.0 (6.80-93.2)	2.87 (0.60-14.2)	0.06 (0.01-0.31)	94.0 (83.5-98.7)
0.31	91.5 (79.6-97.6)	66.7 (9.40-99.2)	97.7 (88.0-99.9)	33.3 (4.30-77.7)	2.74 (0.55-13.6)	0.13 (0.04-0.44)	90.0 (78.2-96.7)
0.32	89.4 (76.9-96.5)	100 (19.4-100)	100 (87.7-100)	37.5 (8.50-75.5)	-	0.11 (0.05-0.24)	90.0 (78.2-96.7)
0.33	83.0 (69.2-92.4)	100 (19.4-100)	100 (86.8-100)	27.3 (6.00-61.0)	-	0.17 (0.09-0.32)	84.0 (70.9-92.8)
**BMI ≤ 28 kg/m2 (n=26)**							
0.30	96.0 (79.6-99.9)	100 (1.30-100)	100 (79.6-100)	50.0 (1.30-98.7)	-	0.04 (0.01-27.3)	96.2 (80.4-99.9)
0.31	92.0 (74.0-99.0)	100 (1.30-100)	100 (78.9-100)	33.3 (1.00-90.6)	-	0.08 (0.02-30.2)	92.3 (74.9-99.1)
0.32	88.0 (68.8-97.5)	100 (1.30-100)	100 (78.1-100)	25.0 (1.00-80.6)	-	0.12 (0.04-0.35)	88.5 (69.8-97.6)
0.33	80.0 (59.3-93.2)	100 (1.30-100)	100 (76.2-100)	16.7 (0.00-64.1)	-	0.20 (0.09-0.44)	80.8 (60.6-93.4)
**BMI > 28 kg/m2 (n=24)**							
0.30	95.5 (77.2-99.9)	50.0 (1.30-98.7)	95.5 (77.2-99.9)	50.0 (1.30-98.7)	1.91 (0.48-7.65)	0.09 (0.01-0.96)	91.7 (73.0-99.0)
0.31	90.9 (70.8-98.9)	50.0 (1.30-98.7)	95.2 (76.2-99.9)	33.3 (1.00-90.6)	1.82 (0.45-7.31)	0.18 (0.03-1.23)	87.5 (67.6-97.3)
0.32	90.9 (70.8-98.9)	100 (9.40-100)	100 (76.2-100)	50.0 (6.80-93.2)	-	0.09 (0.02-0.34)	91.7 (73.0-99.0)
0.33	86.4 (65.1-97.1)	100 (9.40-100)	100 (75.1-100)	40.0 (5.30-85.3)	-	0.14 (0.05-0.39)	87.5 (67.6-97.3)

Se = sensitivity; Sp = specificity; PPV = positive predictive value; NPV = negative predictive value; PLR: positive likelihood ratio; NLR = negative likelihood ratio
